# Methicillin-resistant *Staphylococcus aureus*, Hawaii, 2000–2002

**DOI:** 10.3201/eid1108.050164

**Published:** 2005-08

**Authors:** Fenfang Li, Sarah Y. Park, Tracy L. Ayers, F. DeWolfe Miller, Ralph MacFadden, Michele Nakata, Myra Ching Lee, Paul V. Effler

**Affiliations:** * University of Hawaii School of Medicine, Honolulu, Hawaii, USA;; † Hawaii Department of Health, Honolulu, Hawaii, USA

**Keywords:** Methicillin Resistance, Staphylococcus, Antibiotic resistance, Epidemiology, Surveillance, outpatients

## Abstract

The emergence of methicillin-resistant *Staphylococcus aureus* (MRSA) has generated considerable concern among medical and public health professionals. We used a statewide, population-based antimicrobial resistance surveillance system to examine epidemiologic trends for MRSA from outpatients and inpatients in Hawaii. Pediatric and adult patient populations were compared to assess characteristics of MRSA isolates specific for each group. From 2000 to 2002, 8,206 (26%) of 31,482 total *S. aureus* isolates were MRSA. During this period, the proportion of MRSA isolates increased in both outpatient and inpatient clinical settings (p<0.01). When stratified by age, annual trends showed a significant increase in the proportion of MRSA in adult patients (from 24% to 30%, p<0.01) but not in pediatric patients (from 25% to 27%, p>0.05). Although MRSA isolates from adults demonstrated high resistance to most non–β-lactams, most MRSA isolates from pediatric outpatients remained susceptible to most non–β-lactams.

## Abstract 2

Annual trends showed a significant increase in the proportion of MRSA among adult but not among pediatric patients.

First detected in the 1960s, methicillin-resistant *Staphylococcus aureus* (MRSA) has become the leading cause of nosocomial infections during the last 2 decades (1). MRSA isolates are increasingly resistant to multiple non–β-lactam antimicrobial drugs. Recent reports of vancomycin-resistant *S. aureus* foreshadow an era of chemotherapy in which effective bactericidal drugs to treat infection with this organism may not be readily available (2,3).

Established risk factors for MRSA infection include a history of recent hospitalization or surgery, dialysis, residing in long-term care facilities, presence of an indwelling catheter, and use of injectable drugs (4–6). More recently, however, outbreaks of MRSA infections have been reported in healthy persons without these previously recognized risk factors in a variety of community settings (7–12).

Preliminary work suggests that community isolates of MRSA differ from their hospital counterparts in their demographic, clinical, and molecular characteristics (13–18). However, the epidemiology of MRSA in outpatient settings has not been fully described. In particular, knowledge is limited regarding the epidemiology of MRSA in persons visiting hospital outpatient settings, public or community health centers, and private physicians' offices, i.e., settings where most MRSA infections are treated and the greatest percentage of total antimicrobial use occurs (19). The objective of this study was to better characterize the epidemiology of MRSA from both inpatient and outpatient settings in Hawaii by using a population-based surveillance system.

## Materials and Methods

### Data Collection

Antimicrobial susceptibility test data, collected retrospectively through the State of Hawaii Antimicrobial Resistance Project (SHARP) from 2000 to 2002, were used for this analysis. The SHARP system captures electronic laboratory data from 2 large private clinical laboratories, which serve most of Hawaii's total population (N = 1,211,537) (20). These 2 commercial laboratories provide susceptibility testing services for >85% of all nonhospital outpatient settings in Hawaii (21). They also perform susceptibility testing for 18 of 24 acute care hospitals in the state, including the outpatient services associated with these hospital facilities (22). The remaining 6 acute care hospitals perform susceptibility testing in their own laboratories. Data from 2 of these hospitals are incorporated into the SHARP database, which provides a final dataset that encompasses 20 (83%) of Hawaii's 24 acute care hospitals.

Isolate-level data, including the specimen collection date, source (e.g., blood, urine, and cerebrospinal fluid), susceptibility testing methods (e.g., Kirby-Bauer), and susceptibility test results, are provided by the laboratories participating in SHARP. Limited demographic patient information is also included in the record, e.g., date of birth and sex. However, detailed clinical histories and patient names are not available. In lieu of names, patients were assigned personal identifiers created by concatenating values from the birth date, sex, reporting laboratory, and hospital/clinic location of specimen collection.

All cultures that yielded *S. aureus* isolates from 2000 through 2002 were identified, and any isolates from patients in nonacute care beds (i.e., long-term care homes) and correctional facilities were excluded. Duplicate isolates were then removed according to Clinical and Laboratory Standards Institute (formerly NCCLS) guidelines (23). Only the first isolate per patient, irrespective of body site, antimicrobial susceptibility profile, or other phenotypic characteristics (e.g., biotype), in a defined period (for our purposes, 90 days) was included. Therefore, after the initial isolate, successive isolates for the same patient during the next 90 days were excluded.

The breakpoint for MRSA was an MIC ≥4 μg/mL or a zone diameter ≈10 mm. The breakpoint for oxacillin (methicillin)–intermediate isolates was an MIC 2–4 μg/mL or a zone diameter from 11 to 12 mm. The breakpoint for oxacillin (methicillin)–susceptible isolates was an MIC ≤2 μg/mL or a zone diameter ≥13 mm. Susceptibility interpretations for other antimicrobial drugs were based also on breakpoints established by NCCLS (24).

### Patient Classification

Patients ≤18 years of age were defined as pediatric patients and those ≥19 years of age as adult patients. Classification of inpatient or outpatient status was based on patients' location at the time of specimen collection. Inpatient isolates were defined as those collected from patients in both regular hospital wards and intensive care units. Outpatient isolates were defined as those collected from patients in 1) an outpatient department, emergency department, ambulatory clinic, or same-day-surgery clinic associated with a hospital, or 2) a private physician's office, community public health center, or university health center.

## Data Analysis

The proportion of MRSA was calculated as the number of MRSA isolates divided by the total number of *S. aureus* isolates during a defined period (e.g., per year) for a given setting (e.g., inpatient) and population (e.g., pediatric). The proportion of MRSA isolates resistant to a specific antimicrobial agent was calculated as the proportion of resistant isolates divided by the total MRSA isolates tested against the particular antimicrobial agent of interest during a defined period. Categorical data analysis was performed using EpiInfo version 6.04c statistical software (Centers for Disease Control and Prevention, Atlanta, GA, USA). Chi-square and Fisher exact tests were used to compare proportions, and significance was defined as p<0.05. The Kruskal-Wallis test was used to compare median age of patients, and Pearson correlation coefficient was used to determine the correlation of age and MRSA proportions in different clinical settings.

## Results

A total of 41,250 *S. aureus* isolates were identified from combined inpatient and outpatient settings from 2000 to 2002. After removal of duplicate data, 31,482 isolates remained in the analysis; 23,550 were from outpatients, and 7,932 were from hospital inpatients. A total of 8,206 (26%) of all isolates included in the analyses were MRSA. The overall proportion of MRSA from 2000 to 2002 in outpatients was 22% (5,135/23,550) versus 39% (3,071/7,932) for inpatients (p<0.01). Although the proportion of MRSA remained significantly higher in inpatients compared with outpatients each year of the study, an overall significant increase in the proportion of MRSA infections was observed in both clinical settings during the 3-year study period (p<0.01, Table 1). The proportion of MRSA isolates during the 3-year period was significantly higher in pediatric outpatients (24%, 1,092/4,571) than that in adult outpatients (21%, 4,043/18,979; p<0.01). However, adult inpatients had a significantly higher proportion of MRSA isolates (40%, 2,868/7,217) when compared with pediatric inpatients (28%, 203/715; p<0.01).

**Table 1:** Distribution of methicillin-resistant Staphylococcus aureus (MRSA) isolates by patient group and clinical setting in Hawaii

Year	Patient group	Outpatients			Inpatients		
		No. *S. aureus*	No. (%) MRSA	Contributing to total MRSA (%)	No. *S. aureus*	No. (%) MRSA	Contributing to total MRSA (%)
2000	Total	7,633	1,557 (20)*	–	2,330	842 (36)*	–
	Pediatric	1,593	384 (24)	25	216	65 (30)	8
	Adult	6,040	1,173 (19)	75	2,114	777 (37)	92
2001	Total	7,543	1,495 (20)*	–	2,661	997 (37)*	–
	Pediatric	1,401	302 (22)	20	249	53 (21)	5
	Adult	6,142	1,193 (19)	80	2,412	944 (39)	95
2002	Total	8,374	2,083 (25)*	–	2,941	1,232 (42)*	–
	Pediatric	1,577	406 (26)	19	250	85 (34)	7
	Adult	6,797	1,677 (25)	81	2,691	1,147 (43)	93

*p<0.001, by chi-square test for trend for outpatient and inpatient settings.

**Figure 1:**
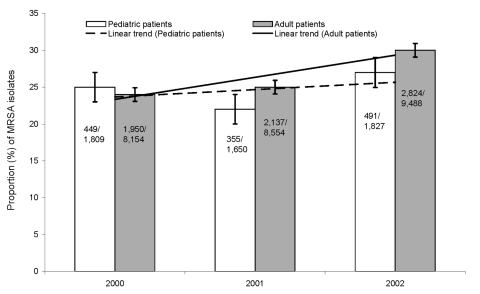
Proportion of methicillin-resistant *Staphylococcus aureus* (MRSA) in pediatric and adult patients, Hawaii, 2000–2002. Error bars show 95% confidence intervals.

**Figure 2:**
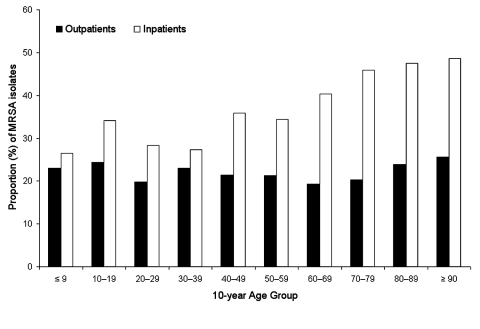
Proportion of methicillin-resistant *Staphylococcus aureus* (MRSA) by 10-year age group and clinical setting, Hawaii 2000–2002.

A significant increase in the proportion of MRSA isolates was observed in all adult patients (from 24% to 30%, p<0.01) during the 3-year study period, and an increasing, although not statistically significant, trend was observed in pediatric patients (Figure 1). When the total MRSA isolates for all study years were examined by 10-year age groups, the proportion of MRSA isolates increased with age in inpatients, but not in outpatients (Figure 2). Correspondingly, inpatients with MRSA infections were significantly older (median age 67 years) than outpatients with MRSA infections (median age 44 years; p<0.01).

Of the 31,482 *S. aureus* isolates in the analysis, 31,240 (99%) had identifiable specimen sources. Table 2 summarizes the frequency of MRSA anatomic specimen sources for pediatric and adult patients in outpatient and inpatient settings. Wounds were the most common specimen sources for MRSA isolates in both pediatric and adult populations in all clinical settings.

**Table 2:** Specimen sources of methicillin-resistant Staphylococcus aureus isolates from pediatric and adult patients by clinical setting, Hawaii, 2000–2002

	Pediatric outpatients (%)	Pediatric inpatients (%)	Adult outpatients (%)	Adult inpatients (%)*
Specimen sources	n = 1,086	n = 199	n = 4,012	n = 2,829
Wound	95	72	80	46
Sputum	0	1	4	29
Urine	2	2	7	8
Blood	0	4	4	7
Other†	3	21	5	9

*Total percentage may not equal 100% because of rounding. †Includes medical devices, respiratory swabs, gynecologic specimens, stool, synovial fluid, and gallbladder specimens.

For both adult and pediatric patients, MRSA resistance to non–β-lactam antimicrobial agents was significantly higher in inpatient isolates than in outpatient isolates for nearly all of the antimicrobial agents tested (p<0.01, Table 3). For all years combined, a significantly higher level of resistance to non–β-lactam antimicrobials was observed in all adult MRSA isolates compared with all pediatric MRSA isolates (p<0.01). Most MRSA isolates from pediatric outpatients remained susceptible to most non–β-lactams, with the exception of erythromycin, to which 24% of the isolates were resistant.

**Table 3:** Resistance patterns of MRSA isolates from pediatric and adult patients by clinical setting, Hawaii, 2000–2002*

Antimicrobial drug	Pediatric patients					Adult patients				
	Outpatients		Inpatients			Outpatients		Inpatients		
	No. isolates tested*	No. (%) resistant	No. isolates tested*	No. (%) resistant	p value†	No. isolates tested*	No. (%) resistant	No. isolates tested*	No. (%) resistant	p value†
Ciprofloxacin	604	11 (2)	110	10 (9)	<0.01	1,502	545 (36)	1,305	1,071 (85)	<0.01
Clindamycin	1,083	71 (7)	202	39 (19)	<0.01	3,936	1,462 (37)	2,830	2,128 (75)	<0.01
Erythromycin	1,092	261 (24)	203	78 (38)	<0.01	4,018	2,186 (54)	2,859	2,412 (84)	<0.01
Gentamicin	997	9 (1)	199	12 (6)	<0.01	3,289	418 (13)	2,609	906 (35)	<0.01
Levofloxacin	215	5 (2)	59	9 (16)	<0.01	629	245 (39)	549	433 (79)	<0.01
Rifampin	1,028	4 (0)	199	5 (3)	<0.01	3,773	134 (4)	2,795	355 (13)	<0.01
Tetracycline	812	27 (3)	178	16 (9)	<0.01	3,414	667 (20)	2,622	976 (37)	<0.01
Trimethoprim/sulfamethoxazole	1,092	1 (0)	203	2 (1)	NS	4,035	199 (5)	2,862	278 (10)	<0.01
Vancomycin	1,092	0 (0)	203	0 (0)	NA	4,036	0 (0)	2,865	0 (0)	NA

*The number of MRSA isolates tested against each antimicrobial drug varies in each laboratory because the drug panel used differed for each MRSA. MRSA, methicillin-resistant *Staphylococcus aureus*; NS, not significant; NA, not applicable. †By chi-square test comparing outpatients to inpatients within each patient group. Fisher exact test was used for rifampin within the pediatric population.

## Discussion

Our analyses identified an increase in the proportion of MRSA for both outpatient and inpatient settings in Hawaii from 2000 through 2002. In the final year of the study, the proportion of MRSA isolates in outpatients and inpatients was notable, i.e., 25% and 42%, respectively.

Whereas high rates of MRSA in inpatient settings have been described previously (1,14), our data highlight an issue of more recent concern, i.e., increasing MRSA infections occurring in the outpatient setting. In the absence of detailed clinical histories, isolates obtained in the outpatient setting cannot be equated to community-associated MRSA. However, isolates from a large population of pediatric outpatients may be a reasonable surrogate for community-associated *S. aureus* infections because they should reflect infections occurring in a predominantly healthy, nonhospitalized population. Furthermore, previous studies have established that skin and soft tissue infections are the most common sources of community-associated MRSA (16,25,26). In our setting, ≈25% of all *S. aureus* infections from pediatric outpatients were MRSA, with 95% of these isolated from wounds. These data suggest that MRSA is an important cause of wound infections in children in Hawaii, a finding consistent with a growing number of reports of MRSA in pediatric outpatients elsewhere (27,28).

Although the proportion of MRSA isolates was significantly lower for adult outpatients compared with pediatric outpatients (21% versus 24%, p<0.01), the total number of MRSA infections in adult outpatients outnumbered those in pediatric outpatients nearly 4:1. In addition, we observed a positive correlation between the proportion of MRSA isolates and advancing age for inpatients, but not for outpatients (R = 0.92, p<0.01). This finding is not unexpected if one considers that older adults are more likely to have chronic illnesses that require more frequent hospitalizations and, thus, potentially encounter increased exposure to resistant microorganisms (1,4,5,14).

MRSA isolates obtained from outpatient settings tended to be less resistant to nonoxacillin antimicrobial drugs than their inpatient counterparts, as observed elsewhere (12,14,16,25–28). Proportionally more pediatric isolates remained susceptible to multiple non–β-lactams compared with adult isolates, most notably when compared with adult inpatient isolates. The dissimilar antimicrobial resistance profiles observed between clinical settings may reflect differences in cohorts of circulating MRSA isolates, especially between outpatients and inpatients, as has been demonstrated in other investigations (16,27,28).

Of clinical importance is that pediatric outpatient MRSA isolates remained largely susceptible to most non–β-lactams. However, ≈25% of pediatric outpatient MRSA isolates in Hawaii were resistant to erythromycin, which is consistent with findings in other studies of MRSA macrolide resistance in pediatric outpatients (25,27,28). In contrast, only 7% of the pediatric outpatient isolates were resistant to clindamycin. This antimicrobial drug has been used to successfully treat MRSA infections in children in community settings. However, clinicians should be aware of inducible resistance to clindamycin due to methylation of ribosomal RNA encoded by the genes *ermA* or *ermC* in initially clindamycin-susceptible but erythromycin-resistant *S. aureus* isolates (25,29). Therefore, clindamycin monotherapy should be avoided in critically ill children with MRSA infections until inducible clindamycin resistance has been excluded by double-disk testing (25,29,30).

A major limitation of this study is the potential misclassification of the clinical setting (i.e., outpatient versus inpatient). Because of the lack of named personal identifiers, patients could not be traced from isolates to be interviewed, and medical records could not be reviewed. Therefore, we could not directly correlate our findings for outpatients and inpatients with the more conventional definitions of community-associated and nosocomial-associated MRSA, respectively. We assigned an isolate to a clinical setting based on the location where the patient's specimen was collected, so misclassification was possible. For example, a patient whose specimen was obtained in an outpatient setting (e.g., an emergency department) may have actually acquired *S. aureus* during a recent hospitalization. If misclassification of this type occurred, the difference in MRSA rates between outpatient and inpatient isolates would be underestimated. However, this misclassification type might be expected to remain consistent over a 3-year period, so the observed temporal trends would likely remain valid. Moreover, the significant differences in resistance to nonoxacillin antimicrobial drugs observed between outpatients and inpatients suggest that the MRSA isolates from outpatients were not, on average, nosocomial-associated isolates cultured in the outpatient setting (13,14,16).

Another study limitation is the inability to fully assess the effect of potential changes in clinical practice during the 3-year study period. If physicians increased or decreased the tendency to obtain cultures, these changes might affect the number or proportion of MRSA isolates identified each year. However, retrospective examination of the total number of cultures requested by physicians each year from 6 acute care hospitals demonstrated that 102,537, 99,772, and 99,461 total cultures were ordered at these facilities in 2000, 2001, and 2002, respectively. These data do not suggest any major shift in practice patterns during the relatively short duration of this study. Therefore, the observed temporal trends cannot likely be attributed to changing physician practices.

A third possible limitation is that we were unable to include susceptibility testing data from 4 of Hawaii's 24 acute care medical centers and their associated outpatient locations. However, a separate review of antibiogram data available from all laboratory facilities in the state in 2001 found that in aggregate, the 4 excluded hospital laboratories accounted for only 1,632 (11%) of the 14,539 total *S. aureus* isolates identified that year. Therefore, the SHARP data in this study captured ≈90% of all *S. aureus* susceptibility testing data in Hawaii from 2000 to 2002.

To our knowledge, this is the first study of MRSA using susceptibility testing data from inpatient and outpatient settings representative of an entire state population. Our major finding, substantive and increasing rates of MRSA in the outpatient setting, is particularly important as reports of MRSA acquired in the community increase across the country, and optimal strategies for the management of MRSA infections in the outpatient setting have yet to be established (14,16,25,27–30). More studies are needed to improve the understanding of MRSA epidemiology in the community and to identify appropriate strategies to prevent *S. aureus* infections, including MRSA.
